# Embryological observations on seed abortion in *Hibiscus syriacus* L. and physiological studies on nutrients, enzyme activity and endogenous hormones

**DOI:** 10.1186/s12870-023-04669-y

**Published:** 2023-12-21

**Authors:** Xiaohong Wang, Jiajia Chen, Lingxuan Hu, Jingwen Zhang, Fen Xiao, Shengqian Zhang, Fengxia Shao, Liqun Huang

**Affiliations:** 1 College of Landscape Architecture, Central South University of Forestry & Technology, Changsha, 410004 China; 2 Hunan Big Data Engineering Technology Research Center of Natural Protected Areas Landscape Resources, Changsha, 410004 China; 3 YeZi Biotechnology company, Yiyang, 413000 China

**Keywords:** *Hibiscus syriacus* L., Seed abortion, Seed set rate, Embryo development

## Abstract

Under natural conditions, most *Hibiscus syriacus* L. individuals form very few mature seeds or the mature seeds that do form are of poor quality. As a result, seed yield is poor and seeds have low natural germinability. These phenomena strongly hinder utilization of the excellent germplasm resources of *H. syriacus*. The study has shown that pollen activity and stigma receptivity were high on the day of anthesis, and the pistils and stamens were fertile. Pollen release and stigma receptivity were synchronous. But in styles following self and cross-pollination, pollen tube abnormalities (distortion and twisting of the pollen tubes) and callose deposition were observed. Cross-pollinated pollen tubes elongated faster and fewer pollen tube abnormalities were observed compared with self-pollinated pollen tubes. And during embryo development, abnormalities during the heart-shaped embryo stage led to embryo abortion. Imbalance in antioxidant enzyme activities and low contents of auxin and cytokinin during early stages of embryo development may affect embryo development. Therefore, a low frequency of outcrossing and mid-development embryo abortion may be important developmental causes of *H. syriacus* seed abortion. Nutrient deficiencies, imbalance in antioxidant enzyme activities, and a high content of abscisic acid at advanced stages of seed development may be physiological causes of seed abortion.

## Introduction

*Hibiscus syriacus* L. is a deciduous shrub or small tree in the Malvaceae family. Approximately 300 cultivars have been bred, which represent a promising pool of germplasm resources. The flower color and shape vary among the cultivars. The flowers are usually large, colorful, and showy, and are borne from June to October [1]. Most accessions have few environmental requirements and are highly tolerant of environmental adversity [2]. As a result, they are commonly used in landscaping as a woody shrub for summer and autumn flowering. In addition, the flowers, leaves, and fruit are used in traditional medicines. The capsule is ovoid and matures in September-November [1]. The mature seeds, which are known as *Chao Tian Zi* in traditional Chinese medicine, are dark brown, kidney-shaped, densely covered with yellowish-white tomentum, and have analgesic and antidotal properties. The reproductive characteristics of *H. syriacus* are mainly self-fertilization, and pollinators are needed for heterosis. Studies have shown that natural bagged seed set of 0.00%, artificial self-pollination seed set of 2.04%, and outcrossed seed set of 35.85% [3]. The seeds produced are mostly empty and shrunken and exhibit low germinability under natural conditions. The poor seed quality and low seed set hinder research on hybrid breeding of *H. syriacus* and impede exploitation of the medicinal value of *H. syriacus* seeds.

The maturation of plant embryos is an important sign of the alternation of sexual and asexual generations of higher plants. However, during sexual reproduction, not all embryos develop in accordance with normal processes leading to formation of a viable embryo [4]. Seed abortion is a widespread phenomenon in nature, causing a significant reduction in crop yield and quality. Crops often fail to form viable seeds owing to seed abortion, which can pose a major obstacle to the selection of improved cultivars. Seed abortion can also severely affect the utilization of superior germplasm resources of ornamental plants. Many factors may cause plant embryo abortion. For example, pollination, fertilization, and embryogenesis may be disrupted at any stage during seed formation and, in addition, the plant’s intrinsic attributes and adverse abiotic factors may cause plant seed abortion.

*H. syriacus* is native to eastern Asia and blooms in summer and autumn. The petals are wide-spreading and the style length is 21.72 ± 1.12 mm. Flowering begins in mid-July and lasts for more than two months. Low seed set under natural conditions; the mature seeds formed are of poor quality, mostly empty and shrunken, with low germinability and a high percentage of seed abortion. The phenomenon of seed abortion not only makes research on *H. syriacus* hybrid breeding more difficult, but also has a certain impact on its seed utilization. It seriously affects the growth, development and application of *H. syriacus*. Therefore, this study mainly studies the internal mechanisms of embryology and physiology in the sexual reproduction process of *H. syriacus*, and systematically explores the reasons for the abortion and low seed setting rate of *H. syriacus* seeds. This research is essential to improve the yield and quality of seeds, increase their utilization value. It also provides scientific basis for further research on *H. syriacus* breeding. Ultimately, the study aims to achieve long-term and effective protection and utilization of this species.

## Materials and methods

### Experimental materials

The study was conducted at the School of Landscape Architecture, Central South University of Forestry and Technology, Changsha, Hunan, China. Healthy, vigorous plants of *H. syriacus* were grown outdoors on the university campus. The average annual temperature in Changsha is 16.8–17.3 °C, the annual sunshine hours are 1500–1762 h, and the average annual precipitation is 1358.6-1552.5 mm. The climate is mild with four distinct seasons, with synchronicity in the abundant precipitation and heat. During the sampling period of July-November in 2021 and 2022, the abundant precipitation and adequate sunshine hours were seasonally typical.

### Assessment of pollen activity and stigma receptivity

Five large unopened floral buds were selected and listed as a reference for sampling. More than 100 large unopened floral buds were selected for emasculating and bagged by enclosing the flower in a sulphate paper bag for subsequent assessment of non-pollinated stigma receptivity. On the day of anthesis, at two-hourly intervals for five consecutive days, 3–5 emasculated bagged and non-emasculated non-bagged flowers at consistent stages of anthesis were sampled. The sampled flowers were transported to the laboratory for assessment of pollen activity and stigma receptivity. Pollen activity was determined using the iodine–potassium iodide (I_2_-KI) staining method [5, 6]. Put an appropriate amount of pollen into a centrifuge tube wrapped in tin foil, add 200 µl I_2_-KI solution to the test tube and shake gently to mix, then place it in the dark for staining for 5 min. Put 2–3 drops of the mixed solution on a glass slide. Select four visual fields under a stereomicroscope to observe and record the stained pollen. Ensure that each visual field contains no less than 200 pollen grains. Set up three biological replicates of the experiment. Pollen that is dyed dark blue or black indicates viability. The benzidine-hydrogen peroxide method was used to estimate stigma receptivity [7]. Place the stigmas and styles of the emasculated flowers on a concave glass slide. Dropwise add the benzidine-hydrogen peroxide reaction solution (with a volume ratio of 1% benzidine: 3% H2O2: water as 4:11:22) to completely immerse the stigmas and styles, and then immediately observe the conditions around the stigmas and styles under a stereomicroscope and capture photos. Examine 3–5 flowers at each time point.

### Observation of pollination, fertilization, and embryo development

Large flower buds were emasculated and enclosed in a sulphate paper bag, then self-pollinated or outcrossed between 08:00 and 10:00 on the day of anthesis. *H. syriacus* cultivar with purple monopetalousa was selected as the pollen donor for cross-pollination. After pollination, the flowers were re-enclosed in a sulphate paper bag. Each pollination treatment comprised more than 200 replicate flowers.3–5 flowers were sampled at 1, 2, 4, 6, 8, 12, 24, 36, 48, and 72 h after pollination and transported to the laboratory. Thereafter, flowers were sampled daily for 1 month after pollination. The flowers were fixed with formaldehyde-ethanol-glacial acetic acid and stored at 4 ℃. Pollen grain germination and pollen tube growth were observed using 1% aniline blue water-soluble fluorescent staining [8]. Thin sections of ovaries (8–12 μm thickness) were prepared using a conventional paraffin sectioning method and stained with hematoxylin-eosin to observe the fertilization process and early embryonic development at 72 h after pollination [9, 10].

### Physiological and biochemical analyses

After pollination, the developing fruit was sampled to observe early embryonic development and every 10 days after the formation of mature embryos. Three to five capsules were sampled at each time point until the seeds within the fruit were mature. One portion of the developing seeds were sampled for measurement of physiological and biochemical indicators. The remaining portion was frozen at -80 °C and was used for determination of phytohormone contents.

The soluble protein content was determined using the coomassie blue staining method [11]. The soluble sugar and starch contents were determined using the anthrone colorimetric method [12]. Superoxide dismutase (SOD), peroxidase (POD), and catalase (CAT) activities were determined with the nitroblue tetrazolium method [13], guaiacol oxidation method [11], and ultraviolet spectrophotometric method [11], respectively. The contents of indoleacetic acid (IAA), abscisic acid (ABA), gibberellin (GA), and cytokinin (CTK) were determined using respective enzyme-linked immunosorbent assay (ELISA) kits (China Enzyme Free Co., Ltd.) following the manufacturer’s instructions. All analyses were conducted with three biological replicates. The data were processed and graphically visualized using Microsoft Excel 2016 and Origin 2022 software, and SPSS 26.0 software was used for significance analysis.

## Results

### Pollen activity and stigma receptivity

**Fig. 1:**
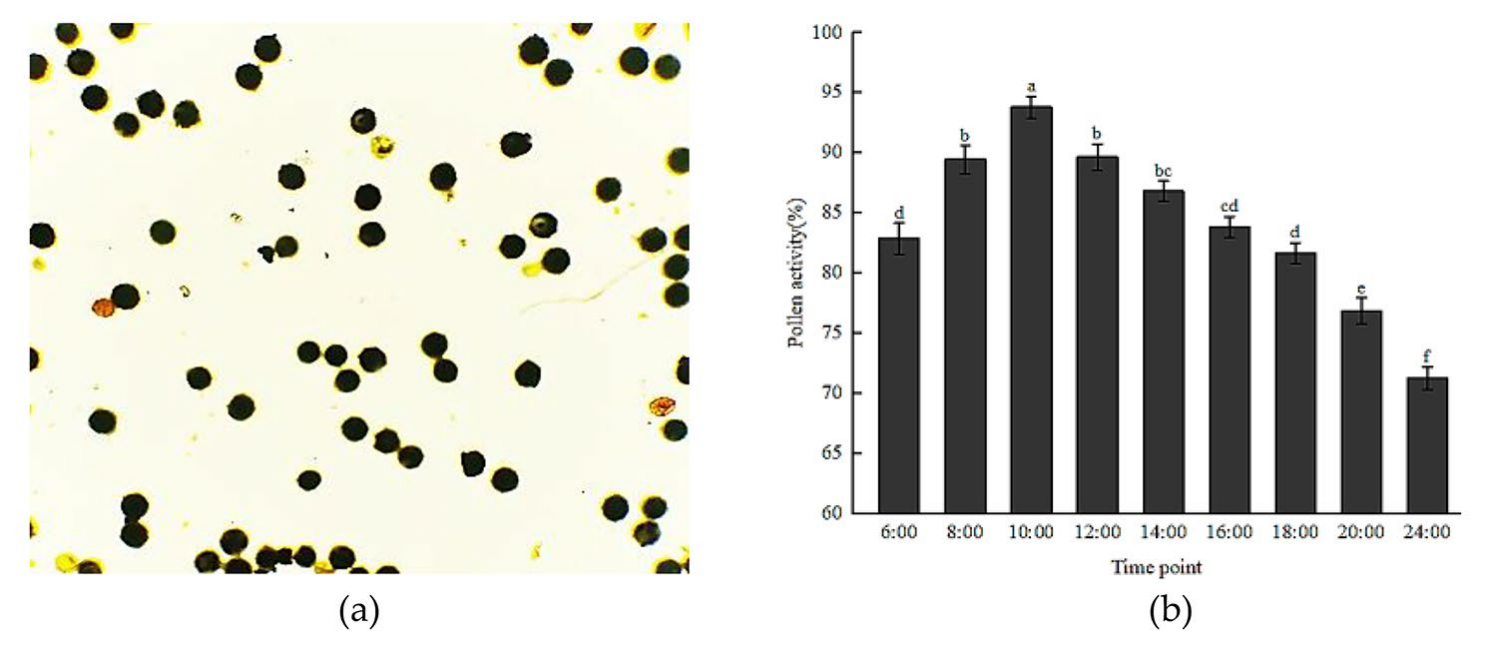
Staining of *H. syriacus* pollen grains with iodine-potassium iodide (**a**) and variation in pollen activity over the course of the day of anthesis (**b**). Bars and error bars indicate the mean ± standard errors (n = 5). Different lowercase letters above bars indicate a significant difference between time points. In SPSS 26.0 followed by the Duncan’s test (*p* < 0.05)

Pollen activity and stigma receptivity both showed an overall trend of initially increasing and thereafter decreasing with time over the course of the day of anthesis (Fig. 1; Table 1). Between 06:00 and 10:00, as the corolla gradually opened, the anthers dehisced to release the pollen grains, and a notable amount of an exudate was visible on the stigma, which enabled adhesion of pollen grains to the stigma. The pollen activity and stigma receptivity both increased continuously during this period. At 10:00, when the single flower is in full anthesis, the pollen activity peaked at 93.82% and stigma receptivity was relatively high. At 12:00 the color of the stigma changed to blue-purple and a large number of air bubbles were produced on the stigma surface, at which point the maximum receptivity of the stigma was attained; the pollen activity was reduced slightly but remained high. At 24:00 the corolla was completely closed and pollen activity declined to the minimum recorded value of 71.24%; the staining intensity of the stigma was the weakest observed and few air bubbles were produced, indicating that the stigma receptivity was at its weakest. On the day of anthesis, the amount of variation in pollen activity was not significant (22.6%), and notable differences in stigma receptivity were observed.

**Table 1:** Stigma receptivity of *H. syriacus* over the course of the day of anthesis, as indicated by benzidine-hydrogen peroxide staining

Time point	Staining intensity
6:00	+/-
8:00	+++/--
10:00	+++/--
12:00	+++/---
14:00	++/--
16:00	++/--
18:00	++/-
20:00	++/-
24:00	+/-

Note: Few bubbles or the appearance of an inconspicuous blue colour is noted as +/-; a large number of bubbles or a distinct blue colour is noted as ++/--; a large number of larger bubbles or the appearance of a blue-purple colour is noted as +++/---

### Pollen grain germination and pollen tube growth

**Fig. 2:**
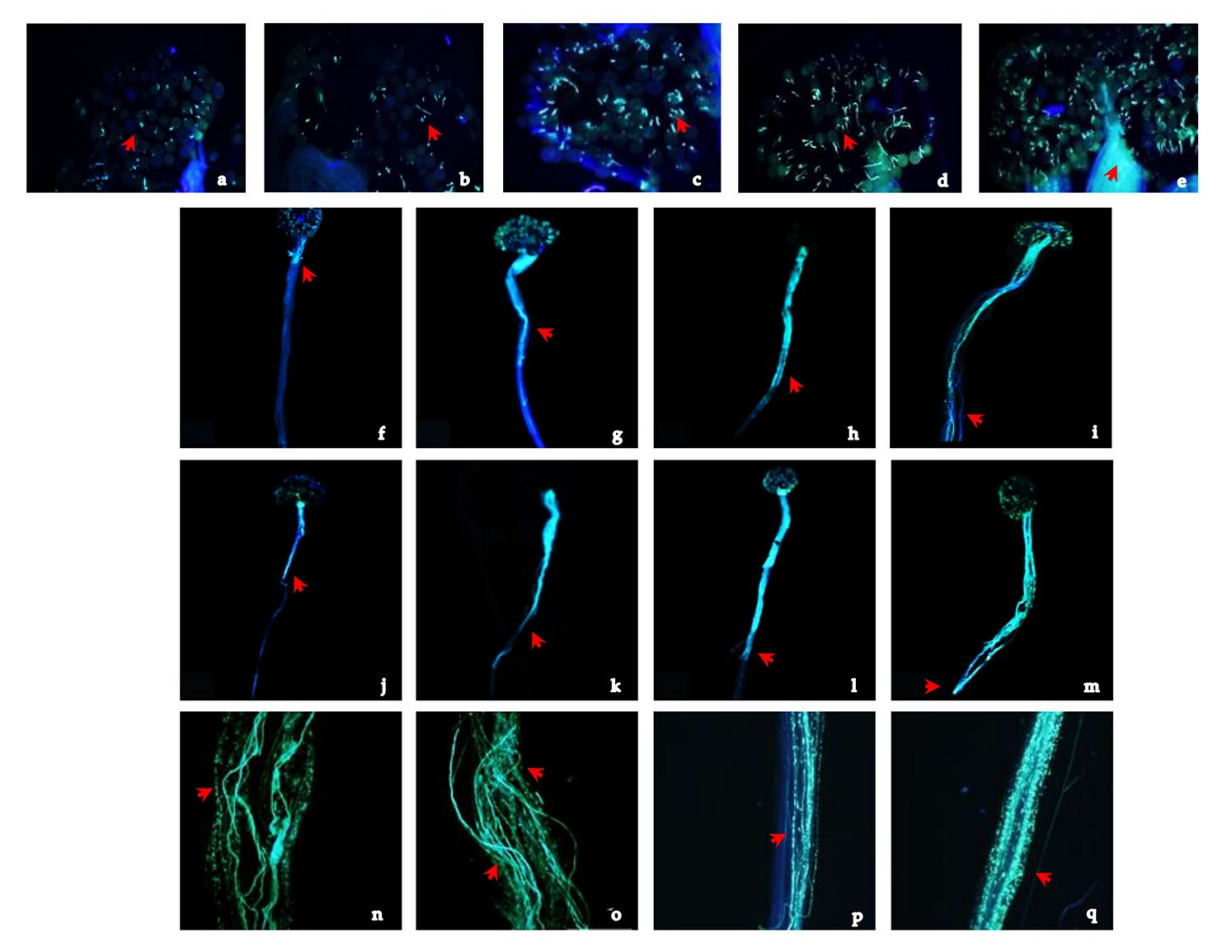
Pollen grain germination on the stigma and pollen tube growth in the style after self-pollination and cross-pollination of *H. syriacus*. **a** ~ **e**: Pollen grains on stigmas at 1, 2, 4, 6, and 8 h after pollination, respectively. Arrows indicate germinated pollen grains. **f** ~ **i**: Growth of pollen tubes in the style at 12, 24, 36, and 48 h after self-pollination, respectively. **j** ~ **m**: Growth of pollen tubes in the style at 12, 24, 36, and 48 h after cross-pollination, respectively. Arrows indicate the tip of the pollen tube in the style. **n** ~ **o**: At 24–48 h after pollination, some pollen tubes were distorted and twisted. **p** ~ **q**: At 24–48 h after pollination, callose had been deposited in the style and at pollen tube apices

Pollen grain germination on the stigma did not differ significantly after self and cross-pollination. At 1 h after pollination, the fluorescent pollen grains were visible on the stigma and a small number of the pollen grains had germinated (Fig. 2a). In most cases, a single pollen tube had emerged from one germination pore. At 2–6 h after pollination, the percentage pollen germination progressively increased (Fig. 2b, c and d). A gradual increase in the number of pollen grains with tubes emergent from two or more germination pores was observed. At 8 h after pollination, almost all pollen grains had germinated, and several pollen tubes had elongated and penetrated the style (Fig. 2e). At 12 h after self-pollination, most of the pollen tubes had elongated for one-third of the length of the style (Fig. 2f). At 24–36 h after self-pollination, the pollen tubes had elongated further down the style, from one-third to half, and from half to two-thirds of the style length (Fig. 2g and h). Distorted, twisted pollen tubes and considerable deposition of callose in the style were frequently observed (Fig. 2n, o, p and q), causing the elongation of some pollen tubes in the style to be halted. At 48 h after self-pollination, the pollen tubes had yet to reach the base of the style (Fig. 2i). At 12 h after cross-pollination, the pollen tubes had elongated to one-third to half of the style length (Fig. 2j). At 24–36 h after cross-pollination, the pollen tubes had elongated from half to two-thirds of the style length to essentially reach the base of the style (Fig. 2k and l). Pollen tube distortion and twisting, and callose deposition were observed in the style during this period (Fig. 2n, o, p and q). However, compared with self-pollinated styles, abnormalities were relatively less frequent in cross-pollinated styles. As a result, a greater number of pollen tubes elongated through the style to the ovary following cross-pollination. By 48 h after cross-pollination, pollen tubes had elongated to the base of the style and had begun to enter the ovary (Fig. 2m).

### Fertilization and embryo development

**Fig. 3:**
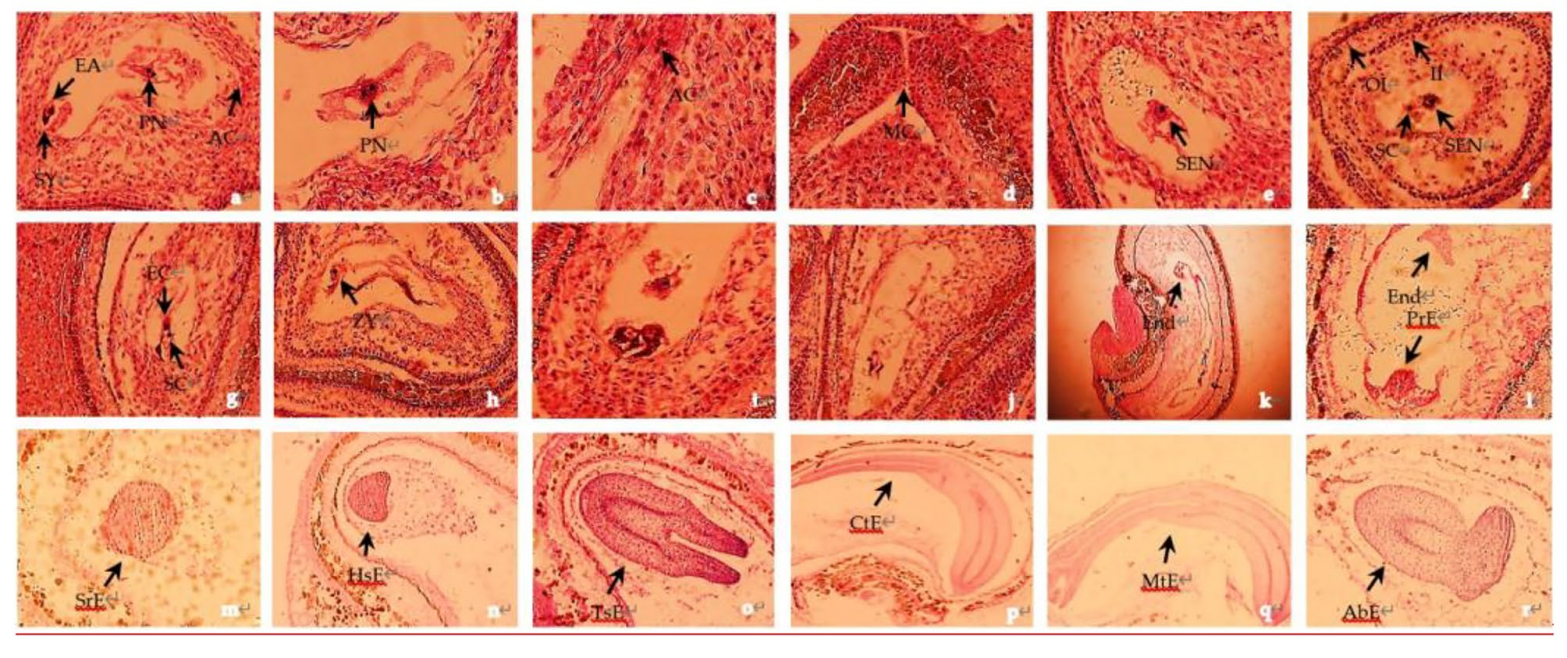
Double fertilization and early embryo development in *H. syriacus*. EA, egg apparatus; SY, synergid; PN, polar nucleus; AC, antipodal cell; SEN, secondary nucleus; SC, sperm cell; EC, egg cell; ZY, zygot; MC, micropyle; OI, outer integument; II, inner integument; PrE, pro-embryo; End, endosperm; SrE, spherical embryo; HsE, heart-shaped embryo; TsE, torpedo-shaped embryo; CtE, cotyledon embryo; MtE, mature embryo; AbE, abortive embryo

The ovary of *H. syriacus* consists of five carpels and five locules, usually bearing six anatropous ovules per locule. The mature embryo sac is formed before the pollen tube enters the ovule. The mature embryo sac is a seven-celled, eight-nucleated structure, with the ovipositor located at the micropylar end consisting of an oocyte and two synergids, with two polar nuclei forming a central cell at the center of the embryo sac, and three antipodal cells situated at the commissure end, arranged in a zigzag pattern (Fig. 3a, b and c). The central cell is connected to the antipodal cells and the ovipositor by intercellular filaments. The periods of pollination and fertilization, embryo sac disintegration, and early embryo development in self and cross-pollinated ovaries were largely, although not entirely, identical. At 4 days after pollination, the pollen tube continued to grow along the inner wall of the ovary, reached the nucellus via the micropyle (Fig. 3d), passed through the nucellus, and then reached the embryo sac and released two sperm cells near the egg cell and the central cell. One of the two sperm cells moved towards the central cell and fused with the secondary nucleus to form the primordial endosperm nucleus (Fig. 3e and f), whereas the other sperm cell moved towards and united with the egg cell to form the zygote (Fig. 3g and h). In some flowers lacking fertilized ovules, the ovary began to change color to yellow and became shrunken at 4 days after pollination, and subsequently abscised as one unit. In other unfertilized flowers, although the ovary did not abscise, paraffin-embedded thin sections showed that the egg cell and synergids in the embryo sac degenerated and the cell structure was blurred, with some of the central cells in the embryo sac still clearly visible or the cells in the embryo sac were completely degenerated and the entire embryo sac cavity was empty (Fig. 3i and j).

Ovules that had completed the double-fertilization process began to expand notably at 5 days after pollination. The zygote became dormant and the primordial endosperm nucleus began to divide to form the free nucleus of the endosperm. After a short period of dormancy, the zygote divided laterally to form a two-celled proto-embryo, which then underwent divisions to form a multicellular proto-embryo, at which time the endosperm nuclei divided vigorously and the free nuclei of the endosperm were concentrated at the chalazal end and the micropylar (Fig. 3k and l). At 9 days after pollination, the terminal cells divided several times to form a spherical embryo (Fig. 3m). At an advanced stage of spherical embryo development, the free nuclei of the endosperm formed cell walls; the resulting endosperm cells were distributed along the inner side of the embryo sac wall. At 11, 13, and 18 days after pollination, a heart-shaped embryo, torpedo-shaped embryo, and cotyledonary embryo were formed, respectively (Fig. 3n, o and p). As the embryo continued to develop, the number of endosperm cells was gradually reduced but the entire embryo was essentially surrounded by endosperm cells. At 21 days after pollination, the mature embryo had formed and filled the entire endosperm sac, and the endosperm had been largely absorbed (Fig. 3q). At an advanced stage of heart-shaped embryo development, some of the embryos developed abnormalities and were malformed, which disrupted the formation of a torpedo-shaped embryo (Fig. 3r).

### Changes in nutrient contents during seed development

**Fig. 4:**
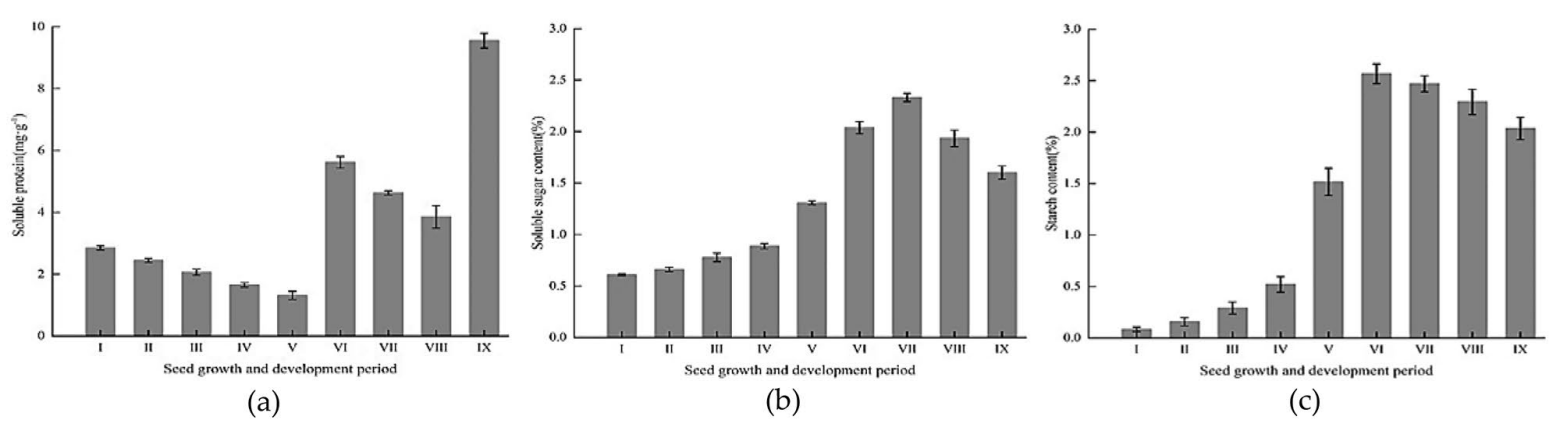
Protein, soluble sugar, and starch contents during seed development in *H. syriacus*. Bars and error bars indicate the mean ± standard errors (n = 3). I, proto-embryonic, 5–8 DAP; II, spherical embryo, 9–10 DAP; III, heart-shaped embryo, 11–12 DAP; IV, torpedo-shaped embryo, 13–17 DAP; V, cotyledon embryo, 18–20 DAP; VI, mature embryo I, 30 DAP; VII, mature embryo II, 40 DAP; VIII, mature embryo III, 50 DAP; IX, mature embryo IV, 60 DAP.

The soluble protein content varied notably during seed development (Fig. 4a), whereas the soluble sugar and starch contents each showed a single-peaked curve (Fig. 4b and c). During early embryo development, the soluble protein content initially decreased and was highest during the proto-embryonic period. As the developing embryo consumed energy, the soluble protein content continued to decrease until a cotyledonary embryo had formed, at which stage the soluble protein content had declined to the minimum value of 1.31 mg/g. Subsequently, in seeds at the mature embryo I stage, the soluble protein content increased rapidly to 5.62 mg/g and thereafter decreased, before increasing rapidly at seed maturity to 9.55 mg/g. The soluble sugar and starch contents were lowest in the proto-embryonic period with mass fractions of 0.61% and 0.08%, respectively. The contents increased slowly in the early stages of embryo development and increased sharply when early embryo development was almost complete, reaching maximum values of 2.33% and 2.57% at the mature embryo II and mature embryo I stages, respectively. Thereafter, the soluble sugar and starch content decreased by 31.33% and 20.71% at the final seed maturity.

### Changes in antioxidant enzyme activity during seed development

**Fig. 5:**
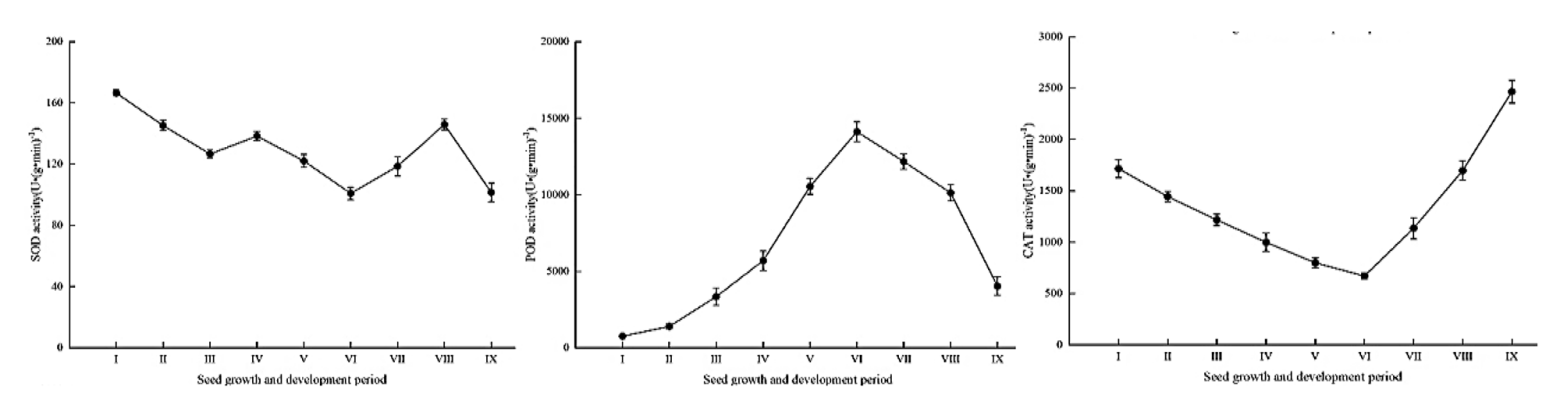
Antioxidant enzyme activities during seed development in *H. syriacus*. Points and error bars indicate the mean ± standard errors (n = 3). CAT, catalase; POD, peroxidase; SOD, superoxide dismutase. I, proto-embryonic, 5–8 DAP; II, spherical embryo, 9–10 DAP; III, heart-shaped embryo, 11–12 DAP; IV, torpedo-shaped embryo, 13–17 DAP; V, cotyledon embryo, 18–20 DAP; VI, mature embryo I, 30 DAP; VII, mature embryo II, 40 DAP; VIII, mature embryo III, 50 DAP; IX, mature embryo IV, 60 DAP.

The SOD activity in the seed showed multiple peaks during seed development, whereas POD and CAT activities each showed a single peak (Fig. 5a, b and c). In the early embryonic period, SOD and CAT activities in the seed reached maximum values of 166.56 U/(g·min) and 955.50 U/(g·min) during the proto-embryonic period, whereas the lowest POD activity was 761.22 U/(g·min). Subsequently, except for a small increase in SOD activity during the torpedo-shaped embryo stage, the overall SOD and CAT activities gradually decreased, whereas POD activity gradually increased until the lowest and highest activities, respectively, were attained at the mature embryo I stage. Subsequently, POD and CAT activities decreased by 71.46% and increased by 72.79%, respectively, during mature embryo development, whereas SOD activity increased to 145.00 U/(g·min) at the mature embryo III stage and decreased again at seed maturity.

### Changes in endogenous hormone contents during seed development

**Fig. 6:**
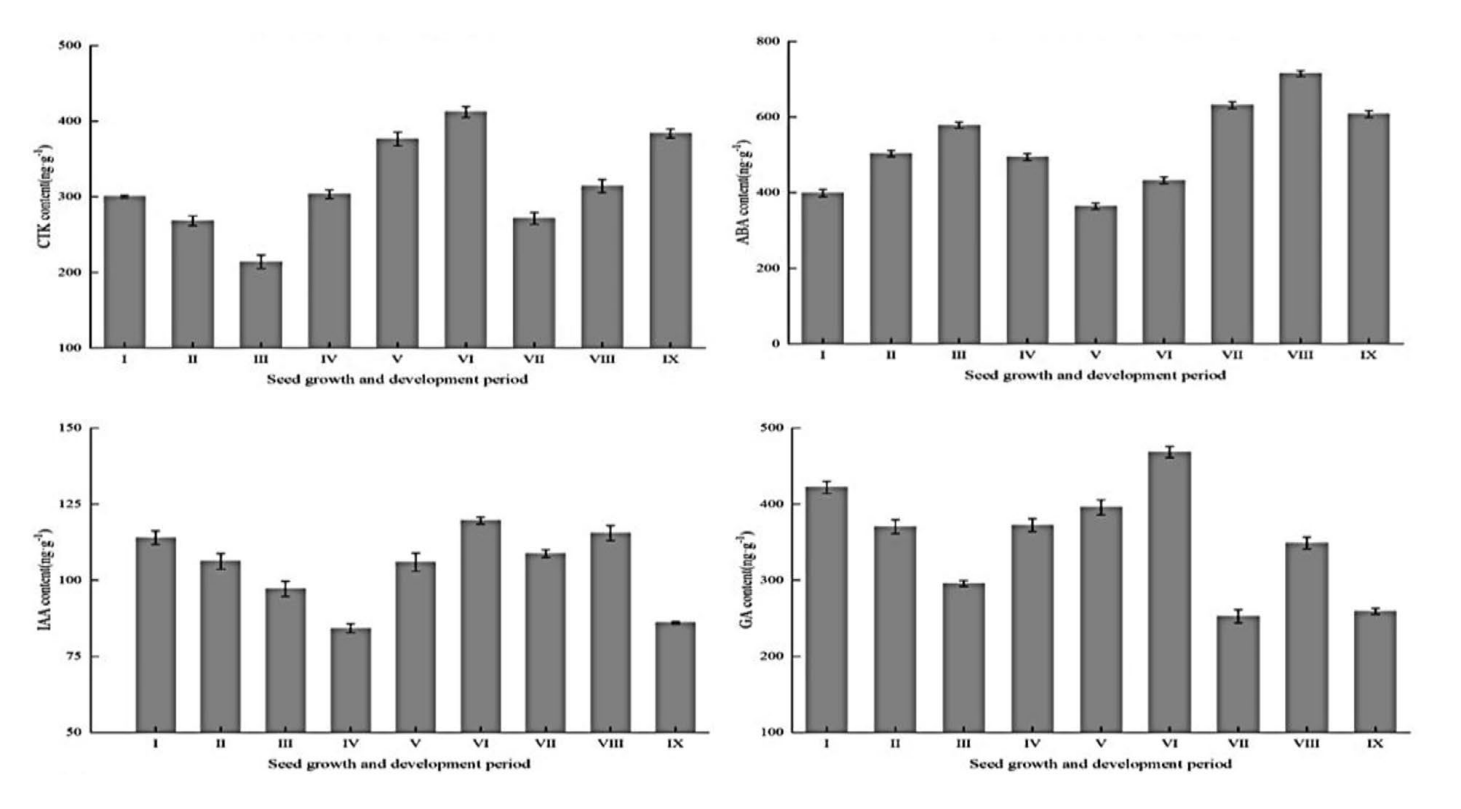
Changes in endogenous phytohormone contents during seed development in *H. syriacus*. Bars and error bars indicate the mean ± standard errors (n = 3). ABA, abscisic acid; CTK, cytokinin; GA, gibberellin; IAA, indoleacetic acid. I, proto-embryonic, 5–8 DAP; II, spherical embryo, 9–10 DAP; III, heart-shaped embryo, 11–12 DAP; IV, torpedo-shaped embryo, 13–17 DAP; V, cotyledon embryo, 18–20 DAP; VI, mature embryo I, 30 DAP; VII, mature embryo II, 40 DAP; VIII, mature embryo III, 50 DAP; IX, mature embryo IV, 60 DAP.

The overall IAA content was relatively low, whereas the ABA content was the highest among the hormones analyzed during seed development. The IAA, GA, and CTK contents in the seed all showed a dynamic trend of initially decreasing and then increasing during the early period of embryo development, whereas the ABA content showed the opposite trend (Fig. 6a, b, c and d). The IAA content gradually decreased to a minimum value of 84.13 ng/g during the proto-embryo to torpedo-shaped embryo developmental period, then increased to a maximum value of 119.63 ng/g at the mature embryo I stage. The trends of changes in the GA and CTK contents were similar. The GA and CTK contents were lowest at the heart-shaped embryo stage at 295.73 ng/g and 213.97 ng/g, respectively; this coincided with the main period of embryo abortion during early embryo development. Subsequently, respective peaks of 468.13 ng/g and 411.93 ng/g were recorded during the mature embryo I stage. The ABA content was low in the spherical embryo period, and then increased to a maximum of 577.93 ng/g in the heart-shaped embryo period, before declining to a minimum of 364.10 ng/g in the cotyledon embryo period. The trends for IAA, GA, and ABA contents were essentially similar in the advanced stages of seed development, with peaks observed in the mature embryo III period. Ultimately, the ABA content decreased slightly as the seeds reached maturity, whereas a more significant decrease of approximately 25% in IAA and GA contents was observed. The CTK content increased gradually again during the mature embryo development period to a peak at seed maturity.

## Discussions

### Pollen viability and stigma receptivity

Pollen viability refers to the ability of pollen grains to germinate and develop a pollen tube, and is an important reference indicator for successful breeding. Stigma receptivity is an important period in the flower maturation process, denoting the duration of the mature stigma’s ability to accept pollen, and is an important determining factor in the percentage seed set after pollination [14]. Pollen viability and stigma receptivity are prerequisites for successful pollination and seed set in flowering plants. Most anthers of a *H. syriacus* flower dehisce and release the pollen grains on the day of anthesis, and thus the amount of available pollen is high. The pollen viability and stigma receptivity of *H. syriacus* were indicated to be high, the pistils and stamens are fertile, and the overall trend for both was to increase and then decrease over the course of the day of anthesis. *H. syriacus* pollen stainability peaked at more than 90% when the petals of the flower had fully expanded, and remained above 70% at all other times on the day of anthesis. The amount of variation in pollen viability on the day of anthesis was not significant. Unlike some plant species, the pollen viability of *H. syriacus* was indicated to remain high throughout anthesis. The duration of pollen viability is strongly associated with species and cultivar [15]. *Lysimachia davurica* pollen loses viability 2 h after flowering [16]. The viability of *Passiflora edulis* pollen peaks at 3 h after flower opening and after 9 h the pollen viability is only 10.63%, thus the duration of pollen viability is relatively short [17]. Similar to *H. syriacus* cultivars ‘Bredon Spring’, ‘Red Heart’, and ‘Russian Violet’ [6], *H. syriacus* shows relatively high stigma receptivity. Between 08:00 and 12:00, the corolla gradually opens, the receptivity of the stigma increases and peaks at mid-anthesis, and thereafter decreases as the corolla closes. At 24:00, the stigma is least receptive but is still fertile. Similar to pollen viability, stigma receptivity is essentially at its highest when the petals are fully expanded but the period of fertility is longer. *Hibiscus syriacus* is similar to *Hesperis oreophila* and most members of the Commelinaceae in that the pollen dispersal and stigma receptivity periods are basically contemporaneous [18, 19]. The corolla is fully expanded when a large quantity of pollen is available for dispersal and the stigma secretes a large amount of exudate. In practical breeding, selection of individual flowers with fully expanded petals for manual self and cross-pollination will greatly improve pollination success and percentage seed set.

### Pollination, fertilization, and embryo development

In angiosperms, abnormalities at any post-pollination stage, e.g., pollen grain germination, pollen tube growth, and post-fertilization embryo development, may result in reduced seed set. At 1 h after pollination, the stigma was already fluorescent, and some pollen grains had started to germinate. At 8 h after pollination, most pollen grains had germinated on the stigma. At 12–48 h after pollination, abnormalities in the growth of pollen tubes, manifested as distortion and twisting, and callose deposition were observed. The pollen tube growth rate and the degree of abnormalities differed between self and cross-pollination. The pollen tube reached the base of the style by 48 h after cross-pollination, whereas after self-pollination this process took longer. Thus, the growth rate of pollen tubes after cross-pollination was greater than that after self-pollination. The pollen tube growth rate of *Camellia oleifera* ‘Huashuo’ and ‘Xianglin XLC15’ is similar to that of *H. syriacus*, and the relative growth rates after self and cross-pollination showed the same pattern. The pollen tubes of *C. oleifera* ‘Xianglin XLC15’ take less time to reach the base of the style compared with those of *C. oleifera* ‘Huashuo’. The cause of this phenomenon may be related to genotype and the temperature after pollination [20, 21]. After self and cross-pollination, the observed abnormalities (distortion, twisting, and callose deposition) caused pollen tubes to aggregate into clumps, and led to some pollen tubes growing upwards or ceasing growth, and thus failed to enter the embryo sac to effect fertilization, making it difficult to form embryos. This phenomenon has also been observed in *Rhododendron simsii* [22], *Lilium brownii* [23], *Rosa persica* [24], and *Pyrus* spp. [25]. Pollen tube aberrations are a common reason for failure of ovules to be fertilized and are a primary cause of pre-fertilization disorders and low fertility in plants. Pre-fertilization disorders, such as apical bending, central dilation, and callus deposition, in tropical and hardy *Nymphaea tetragona* hybrids severely impede pollen tube entry into the ovule [26]. In *Dianthus chinensis* hybrids, large callose masses hinder pollen tube growth, in addition to abnormal accumulation and expansion of callose at the tip of the pollen tube, which are among the important factors responsible for low percentage seed set [27].

However, the seed set of plants is not only related to their own characteristics, but also to the external environment and other factors. In the present study, it was observed that when pollination was performed at temperatures above 37 °C, the ovaries began to wilt and abscise at 4 days after pollination. Most *H. syriacus* accessions have difficulty in setting fruit during the summer blooming period, and the ovaries began to yellow and abscise 4–5 days after the day of anthesis. It is unknown how temperature affects pollination and fertilization in *H. syriacus*. However, a previous study has reported that high temperature reduces the growth rate of *Gossypium hirsutum* pollen tubes [28]. Unsuitable temperatures can lead to stagnation of pollen tube growth in *Citrus clementina*, thus affecting the number of pollen tubes reaching the ovary [29].These studies provide potential ideas for future studies to systematically and comprehensively analyze the causes of low seed set in *H. syriacus*. The present results revealed that abnormalities in pollen tube growth are an important cause of pre-fertilization disorders in *H. syriacus*, leading to fruit abortion, and may be an important factor affecting the seed set of *H. syriacus*.

Embryo development in *H. syriacus* is similar to that of dicotyledonous plants in general, passing through the stages of protoembryo, spherical embryo, heart-shaped embryo, torpedo-shaped embryo, cotyledonary embryo, and mature embryo. The mature embryo was formed by 21 days after pollination. Abnormalities in early embryo development of *H. syriacus* were observed and embryo abortion mainly occurred during the development of a heart-shaped embryo. The abnormal degradation and stagnation of embryo development after fertilization are the main causes of embryo abortion mid-development [30], and mid-development embryo abortion is a major cause of seed abortion. Post-fertilization embryo abortion was also a cause of seed abortion in *H. syriacus* and contributed to the low percentage seed set. Cessation of embryo growth at the cotyledonary embryo stage in *Syringa microphylla* leads to seed abortion [31]. A higher frequency of embryo abortion was observed in crosses between *Chrysanthemum morifolium* and *Ajania przewalskii*, resulting in low percentage seed set [32]. Among the main causes of seed abortion in crosses between the wild tomato species *Solanum peruvianum* and *S. chilense* is stalled embryo development. Several factors may cause plant embryo abortion. *Ziziphus jujuba* ‘Jinsixiaozao 11’ and ‘Wuhexiaozao 72’, and *Osmanthus fragrans* are subject to abnormal embryo development owing to early degeneration of the embryo and endosperm [33, 34]. However, many endosperm cells remain around the abnormally developed heart-shaped embryo in *H. syriacus*. The primary cause of embryo abortion in *H. syriacus* is unclear and may be associated with physiological properties, such as nutrient contents, enzyme activities, and endogenous hormone contents, in the ovule.

### Physiology and biochemistry of seed development

Nutrients from the maternal parent are stored in the endosperm and are an important source of nutrients for seed formation, not only for embryo development but also for carbohydrate metabolism, storage, and transport [35]. During early embryo development, protein and sugars are the principal sources of energy. As *H. syriacus* seed development continued, the soluble protein content declined to a minimum during the cotyledonary embryo stage. This trend was exactly opposite to that reported for sunflower (*Helianthus annuus*) seeds in which protein, the main nutrient in the seed, increases in content as the seed develop to maturity [36]. The soluble sugar content then increases rapidly as the cells proliferate and divide, and starch is accumulated. The interconversion of starch and sugars provides energy-storage material for the seed, which in turn acts as a source of energy for seed maturation. The soluble sugar and starch contents of the seeds of *Nelumbo nucifera* are similar to those of *H. syriacus*, continuing to increase as the embryo differentiates and develops, and the soluble sugar content is higher than the starch content, enabling the accumulation of starch [37]. The source of nutrients and energy required for advanced seed development is usually provided by sugars stored in the endosperm and embryo, but the endosperm cells of *H. syriacus* degenerate after the formation of the mature embryo and the nutrient content is markedly reduced. The lack of nutrients may affect seed development, which supports the statement by He et al. that nutrient deficiency may be the cause of the low seed set in *H. syriacus* [38]. In aborted ovules of *Castanea henryi*, only a small amount of starch is present, whereas normally developed ovules are smooth and store abundant starch; the lack of starch leads to abnormal embryo development, resulting in embryo abortion [39]. Wang observed that inadequate nutrient (i.e., sugar) supply to the endosperm can lead to severe seed abortion in *Glycine max* [40]. The limited nutrient content of *H. syriacus* seeds at advanced stages of development, coupled with a high number of developing seeds, will result in the availability of fewer nutrients for individual seeds, and thus may result in formation of poor-quality seeds or seed abortion in the advanced stages of seed development, thus affecting the percentage seed set.

The activities of SOD, POD, and CAT reflect the severity of stress to which plants are subjected. The three enzymes act synergistically to maintain stable concentrations of free radicals, preventing oxidative damage to cell membranes, and thus assist in maintaining homeostasis. The rapid growth of seeds during early embryo development accelerates metabolism in the seed and generates a large quantity of free radicals. At this stage in *H. syriacus*, SOD and CAT activities in the seed remained relatively high, but POD activity was low, which may have difficulty in scavenging excessive H_2_O_2_, causing accumulation of H_2_O_2_ and damage to the structure of cell membranes, leading to embryo abortion. In the same period, a transient and rapid increase in SOD activity from the heart-shaped embryo to the torpedo-shaped embryo stages was observed, and embryo development was abnormal during this period. Principal component analysis and gray correlation analysis revealed that ovule SOD activity was the most important factor influencing the abortion of seedless grape embryos [41]. Thus, there may be a relationship between embryo abortion and abnormal enzyme activity. At advanced stages of seed development, reduced water content causes a rapid increase in SOD and CAT activities to resist external stresses, which leads to accumulation of various reactive oxygen species, but POD activity is again reduced. Low activity of protective enzymes may lead to loss of cell membrane function and metabolic disorders, resulting in seed abortion [42]. The POD and CAT activities of *Cypripedium japonicum* seeds are reduced to a minimum at advanced stages of seed development, resulting in low activities of protective enzymes and disruption of cell-membrane structure at seed maturity, which may lead to disrupted cell structure and seed abortion [43]. Therefore, the relatively low activity of protective enzymes at advanced stages of seed development may also contribute to seed abortion in *H. syriacus*.

Endogenous hormones, as important regulators of seed development, are present in low quantities but regulate the physiological state and biochemical changes throughout seed development, contributing to the increase or decrease in intensity of the seed’s vital activities. In the early stages of seed development, when embryo metabolic activity is high, seeds contain relatively high contents of growth-promoting hormones, which promote cell division, and low levels of inhibitory hormones. The contents of CTK and GA declined sharply during the main period of embryo abortion, whereas ABA contents rose rapidly, in *H. syriacus*. Insufficient supply of growth-promoting substances may affect cell division and the synthesis of biomolecules in the embryo, resulting in disruption of physiological metabolism, and energy deficits, abnormal embryo development, and eventually leads to embryo abortion. At the same time, low contents of IAA and CTK are not conducive to seed development and the formation of full seeds [44]. Chen observed that a dramatic decrease in CTK content may inhibit cell division and block nucleic acid and protein synthesis in early-stage embryos, leading to embryo abortion in *Litchi chinensis* ‘Lanzhu’ [45]. Therefore, IAA, GA, and CTK promote seed expansion during early embryo development by facilitating cell differentiation and elongation, and embryo abortion may also be correlated with hormone content. At advanced stages of seed development, IAA promotes growth by regulating the synthesis of nucleic acids and proteins, and attracts assimilates for transport from the leaves to the seeds; GA promotes the synthesis of nutrients, such as starch, allowing the seeds to continue to develop [46]. Growth-promoting hormones are involved in nutrient transport and synthesis, thereby promoting seed development and seed maturation. Concurrently, the rapid rise in ABA content reaches a peak, which would not only promote the interconversion and accumulation of nutrients during embryo development, but also promote seed maturation and abscission. However, high contents of ABA may inhibit cell division and differentiation, nutrient input, and biomolecule synthesis, putting seeds at a disadvantage in competition for resources, eventually leading to a lack of nutrients and cessation of development [47]. The ABA content is high in moderately deficient versus highly deficient *Eriobotrya japonica* seeds with delayed development [48].Therefore, high contents of ABA at advanced stages of seed development may also inhibit nutrient synthesis, leading to nutrient deficiencies and resulting in seed abortion.

## Conclusions

In summary, pollen stainability and stigma receptivity are high on the day of anthesis, and the pistils and stamens are fertile in *H. syriacus*. The duration of high pollen stainability and stigma receptivity is prolonged and synchronous. The optimal time for pollination is between 08:00 and 12:00 on the day of anthesis. Choosing this time for pollination will effectively increase the success rate of pollination and fertilization, thus improving the percentage seed set. A study of the reproductive biology of *H. syriacus* revealed that poor pollination and embryo abortion are among the most important causes of seed abortion in *H. syriacus*, and may also be the main cause of seed abortion in *H. syriacus*. Nutrient deficiencies, imbalance in antioxidant enzyme activities, and high contents of ABA during seed development may also cause seed abortion, thus affecting fruit set. The present results provide practical guidance and a theoretical basis for pollination and genetic breeding of *H. syriacus*, as well as data on the reproductive biology, and the physiology and biochemistry of seed development, of *H. syriacus*. Many factors affect plant seed development and percentage seed set, and external environmental influences as well as gene-level regulation may impact on these. The present study provides ideas for future research into the causes of the low percentage seed set in *H. syriacus* cultivars.

## Data Availability

All data sheets and codes to process data are available upon request to the corresponding author, Xiaohong Wang (t19960222@csuft.edu.cn).
